# Renal Cell Carcinoma with Synchronous Metastasis to the Calcaneus and Metachronous Metastases to the Ovary and Gallbladder

**DOI:** 10.1155/2011/671645

**Published:** 2011-10-19

**Authors:** Jasper Decoene, Filip Ameye, Evelyne Lerut, Raymond Oyen, Hein Van Poppel, Steven Joniau

**Affiliations:** ^1^Department of Urology, Düsseldorf University Hospital, 40591 Düsseldorf, Germany; ^2^Department of Urology, University Hospitals KULeuven, 3000 Leuven, Belgium; ^3^Department of Histopathology, University Hospitals KULeuven, 3000 Leuven, Belgium; ^4^Department of Radiology, University Hospitals KULeuven, 3000 Leuven, Belgium

## Abstract

Renal cell carcinomas (RCCs) are known for their unpredictable metastatic pattern. We present the case of a 63-year-old woman who initially presented in 1992 with a metastasis in the left calcaneus that led to the discovery of RCC. In 1998, a new metastasis was found in the ovary. In 2008, the diagnosis of a gallbladder metastasis was made. All metastases were surgically removed; no additional systemic therapies were used. Aggressive surgical treatment can prolong the survival of patients with resectable metastases. Patterns of metastasis are discussed, and a brief review of the literature is given regarding each localization.

## 1. Introduction

In 2010 kidney cancer accounted for 4% and 3% of all newly diagnosed malignancies in men and women, respectively [1]. From 80% to 85% of these cancers are renal cell carcinomas (RCCs), which are often diagnosed during radiographic examinations for other purposes. At the time of diagnosis, approximately one-third of patients present with metastatic disease. The outcome in patients with metastatic disease is very poor, with a 5-year survival rate of only 5% to 10% [2].

## 2. Case Report

**Figure 1:**
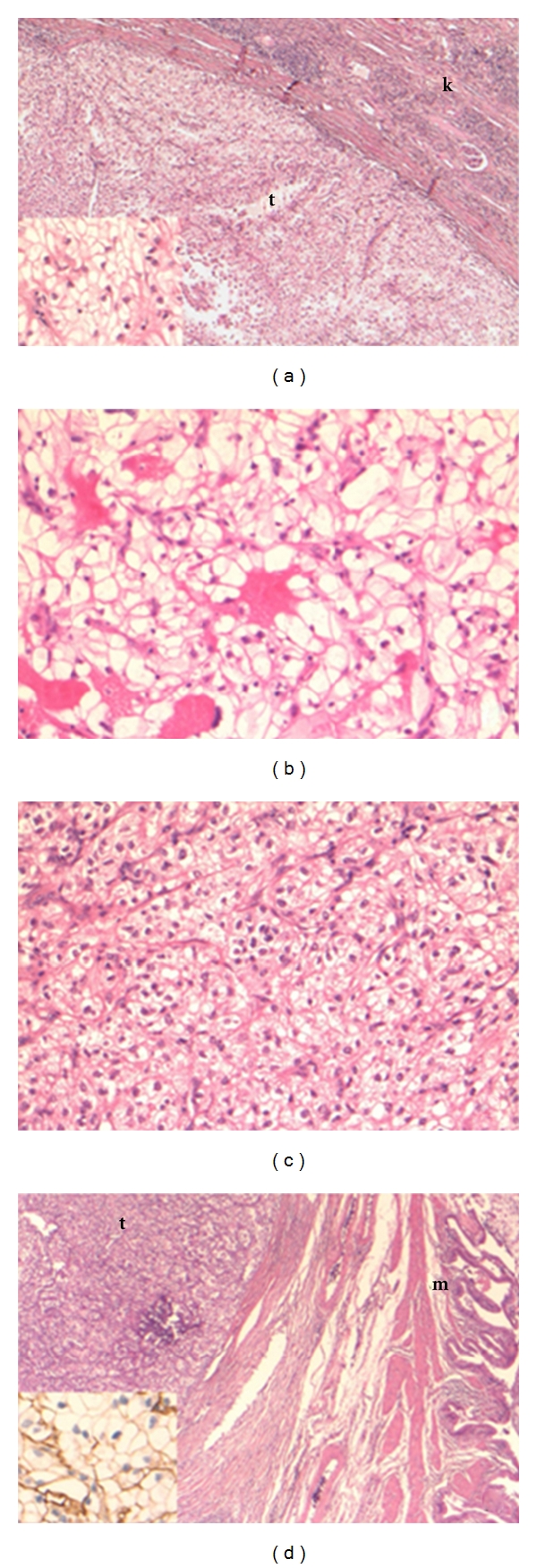
(a) Primary renal cell carcinoma (clear cell type) in the kidney. The tumor (t) is sharply demarcated from the renal parenchyma (k). The tumor consists of sheets of large epithelial cells with an optically empty cytoplasm and sharp cell borders (inset). (original magnification 25x and 200x (inset); Hematoxylin Eosin stain), (b) Metastasis of clear cell carcinoma in the oscalcaneum. Note the similar cell type in metastasis and primary tumor (original magnification 200x; Hematoxylin Eosin stain). (c) Ovarian metastasis of clear cell carcinoma. Note the similar cell type in metastasis and primary tumor (original magnification 200x; Hematoxylin Eosin stain). (d) Metastasis of clear cell carcinoma in the gallbladder. The tumor (t) is located deep in the cholecystic wall (m: mucosa) (original magnification 25x; Hematoxylin Eosin stain). Inset: the tumor cells show the typical membranous staining pattern for CD10, consistent with the immunophenotype of a clear cell RCC (original magnification 200x; immunohistochemical CD10 stain).

In 1992, a 47-year-old woman presented with left heel pain. After further examination, the orthopedic surgeon identified a possible malignancy of the calcaneus. After wedge excision, histopathology identified the lesion as a metastasis originating from an RCC (Figure 1). Further radiographic investigations revealed RCC in the right kidney. Adjuvant radiation therapy of the calcaneus and right radical nephrectomy followed. Histopathology of the kidney showed a tumor of 5 cm, consisting of a clear cell adenocarcinoma Führman grade 3. Vascular invasion, but no capsular extension, was observed (Figure 1).

**Figure 2:**
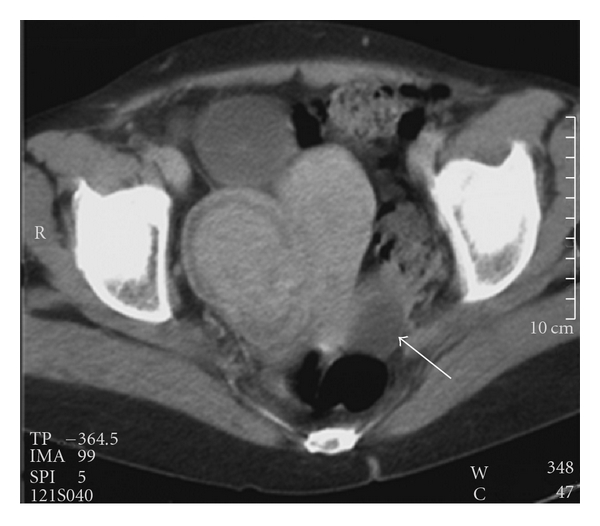
Contrast-enhanced CT of the pelvis showing a large myoma on the right side of the uterus. Note the cyst-like lesion in the left ovarium, without intraluminal nodules (arrow).

For the next 5 years, followup showed no recurrences. Nevertheless, ultrasonography in December 1997 revealed a large polylobular mass in close connection with the uterus. Tomography revealed multiple adenopathies and a fibromyomatous uterus (Figure 2). Hysterectomy, bilateral ovariectomy, and iliaclymphadenectomy were performed. Pathologic analysis showed a uterine myoma, a negative right iliac lymph node, and a clear cell tumor in the left ovary compatible with an RCC metastasis (Figure 1). 

**Figure 3:**
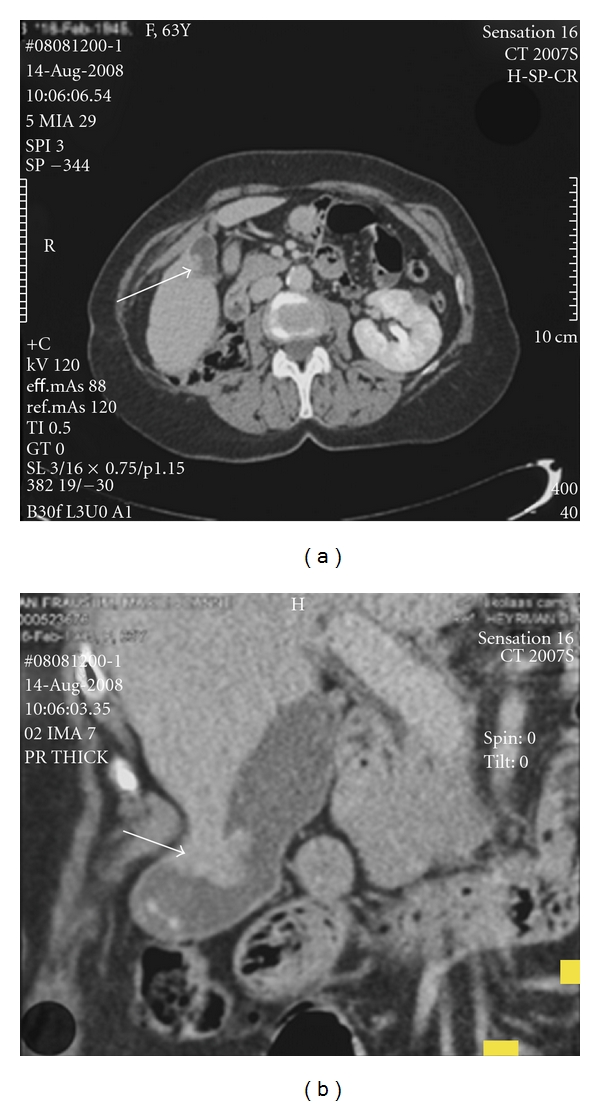
CT scan showing status after right nephrectomy and an evolutive papillary lesion in the gallbladder of approximately 1.9 cm ((a) and (b), arrows). (b) Contains the coronal reconstruction.

For the next 11 years, radiographic followup was uneventful. In August 2008, an abdominal CT scan identified a polypoid lesion in the gallbladder (Figure 3). A laparoscopic cholecystectomy was performed. Macroscopic and microscopic appearances led to the diagnosis of an RCC metastasis (Figure 1).

## 3. Discussion

Kidney cancer is one of the most deadly urological tumors. The 5-year relative survival rate for all stages is approximately 69.5% [3]. At initial diagnosis, one-third of patients present with metastasis [2]. According to the study of Lam et al., eventually, up to 28% of patients with clinically localized disease develop distant metastatic disease within 5 years [4]. Diagnosis of metastases precedes RCC diagnosis in only 5% of cases.

The most frequent localizations, in order of frequency, are the lungs, bones, liver, lymph nodes, adrenals, and brain. However, RCC metastases have been described in virtually every organ of the human body [5].

In the case of bone metastases, the spine (80% of localizations) and the long bones (10%) are most commonly involved; the distal bones of the hands and feet are very rarely involved. One study that examined 2800 bone tumors found only 19 in the foot, of which 11 were metastatic. The calcaneus is the most frequently affected bone in the foot, followed by the tarsal bones. Metastases of RCC to the calcaneus are very rare. They are associated with a poor prognosis, due to their relationship with diffuse metastatic disease [6, 7].

Kollender et al. concluded that aggressive surgical excision of RCC bone metastases is justified for pain relief, local tumor control, and the prevention of morbidity associated with pathological fractures. Although the patient population was rather small, a relatively prolonged survival was shown [8]. Patients also have a significant better survival if a tumor-free resection margin can be achieved. If a complete resection of multiple metastases (even a combination of osseous and visceral metastases) is technically feasible, 5-year survival rates up to 40% are possible [9].

In our case radiotherapy was performed after the surgical excision of the calcaneal metastasis. Despite the general accepted fact that the renal cell carcinoma is radioresistant, several study groups have clearly showed a palliative benefit in symptomatic osseous metastases from these tumors. None of the mentioned reports investigated possible survival advantages [10–12]. 

Ovarian metastases originating from RCC are very rare and to the best of our knowledge have only been described in few cases. Valappil et al. listed 12 cases, and Insabato et al. described another 3 cases. Involvement of the contralateral ovary is even rarer. In most of the cases, the ovarian mass was detected after the diagnosis of the renal tumor. RCC metastases in the ovaries are often misdiagnosed as primary ovarian clear cell carcinomas, due to the presence of clear cells in ovary tumors as well as in RCCs. With immunohistochemical techniques, the ability to differentiate between these tumors is improving [13–15].

Gallbladder metastases due to primary RCC are rare. In the report of Ishizawa, covering a period of more than 50 years, 24 cases have been described. Almost all patients were men. In the present case, the time interval between primary tumor and gallbladder tumor is 16 years; only two authors have reported longer time intervals (27 years in both cases). In almost all cases, the gallbladder tumor was found in routine followup, without any associated symptoms. Followup in this group of 24 patients showed a maximum tumor-free period of 6 years after cholecystectomy. However, late recurrence of RCC can never be excluded [16].

In metastatic renal cell carcinoma patients, the use of molecular targeted therapies that block the VEGF pathway or the mTOR pathway offers new perspectives. For the moment, different molecules are available, each with their own efficacy and side effects. Clinical guidelines for the first- and second-line treatment exist, but the real question lies in the individual treatment of each patient. There is a need for prognostic factors and biomarkers. Better results may be achieved by using combination or sequential therapy. And the search for other pathways and new agents interfering in these pathways should continue [17].

Several studies have shown metastasectomy for resectable tumors to result in increased survival compared to no surgery. Especially in the case of lung metastases, 5-year survival rates of up to 50% have been reported [18]. As mentioned before, a complete resection of bone metastases can result in a 5-year survival rate of 40% [9]. Patients with brain metastases tend to have a poor prognosis, due to the fact that often other extracranial regions are involved. Wroński et al. analysed 50 operatively treated patients, with a median survival of 12.1 months from the diagnosis of the brain metastases [19]. The biggest series on metastasectomy for liver metastases from RCC included 31 patients and showed 5-year survival rates of 38.9% [20]. Molecular targeted therapies may provide a useful tool in combination with surgical removal of metastases. A recent study showed the feasibility of this approach with acceptable morbidity. A longer progression-free survival could be reached, but further investigations remain necessary [21].

## 4. Conclusion

Given the poor 5-year survival in patients with metastatic RCC, it is rather exceptional that our patient is still alive 17 years after the initial RCC diagnosis. This woman has already presented with different metachronous metastases, emphasizing the importance of a strict radiographic followup. The use of radiotherapy in the case of osseous metastases is reserved for the palliative setting. We do not plead against the use of targeted therapies, but when technically feasible surgery should play an important role. Thanks to aggressive treatment of each metastasis, the life expectancy of our patient remains higher than the average life expectancy patients with metastatic RCC would indicate.
